# Early Intervention for Children With Developmental Disabilities and Their Families via Telehealth: Systematic Review

**DOI:** 10.2196/66442

**Published:** 2025-01-17

**Authors:** Yoomi Shin, Eun Ju Park, Anna Lee

**Affiliations:** 1 College of Nursing and Brain Korea 21 FOUR Project Yonsei University Seoul Republic of Korea; 2 College of Nursing Yonsei University Seoul Republic of Korea; 3 Mo-Im Kim Nursing Research Institute College of Nursing Yonsei University Seoul Republic of Korea; 4 Institute for Innovation in Digital Healthcare Yonsei University Seoul Republic of Korea

**Keywords:** developmental disabilities, developmental delay, early intervention, telehealth, digital intervention, autistic spectrum disorder, cerebral palsy, family-centered care, multidisciplinary care, systematic review

## Abstract

**Background:**

Early intervention during the first 3 years of life is crucial for children with developmental disabilities to optimize developmental outcomes. However, access to such services is often limited by geographical distance and resource constraints. Telehealth can be part of a solution for overcoming these barriers, enabling the delivery of early intervention services. However, a comprehensive understanding of the efficacy and implementation of telehealth in early interventions remains elusive, particularly for children aged 0-3 years.

**Objective:**

This systematic review aims to synthesize existing research on the effectiveness and implementation of telehealth interventions in infants and toddlers (aged 0–3 years) who are at risk of or diagnosed with developmental disabilities. The primary objective of the study is to evaluate the ways that telehealth compares to conventional in-person interventions in improving developmental outcomes for children and supporting family well-being.

**Methods:**

A systematic search was conducted of 4 electronic databases (PubMed, Embase, CINAHL, and Web of Science), focusing on studies published between 2010 and 2024. The inclusion criteria were studies involving telehealth interventions for children aged 0-3 years who were at high risk or had developmental disabilities, which involved active interactions between the providers and the families. Study quality was assessed using the mixed methods appraisal tool, and a narrative synthesis was used to analyze the data.

**Results:**

Eighteen studies met the inclusion criteria: 12 single-case designs, 4 randomized controlled trials, and 2 nonequivalent control group designs. All studies involved caregiver-child dyads, with child ages ranging from 5 to 37 months and having or at risk of autistic spectrum disorder (n=10, 56%), cerebral palsy (n=4, 22%), and other conditions (n=4, 22%). Synchronous videoconferencing was the primary modality for caregiver training and coaching (n=17, 94%) while 1 intervention used an Internet of Things system. Outcomes were identified in child communication (n=9, 50%), physical (n=6, 33%), social or emotional (n=6, 33%), and adaptive behavior (n=4, 22%), as well as caregiver implementation (n=12, 66%). Telehealth demonstrated comparable or superior effectiveness to traditional in-person methods in 2 studies. However, the focus on specific conditions and limited research on cognitive development were notable gaps.

**Conclusions:**

Telehealth can be a viable alternative to traditional in-person early interventions for young children who have developmental disabilities and their families. It enhances accessibility and interactions between families and providers at a distance while promoting family-centered care. Challenges exist, including those of technological literacy, and the lack of research on cognitive outcomes must be addressed. Future work should explore more comprehensive interventions, including multidisciplinary approaches and expanded family outcomes, to solidify the role that telehealth plays in early intervention.

**Trial Registration:**

PROSPERO CRD42024551286; https://www.crd.york.ac.uk/prospero/display_record.php?RecordID=551286

## Introduction

Child mortality rates have decreased dramatically in recent decades, but the prevalence of developmental disabilities in children is gradually increasing [1,2]. Developmental disabilities are a group of conditions due to an impairment in physical, learning, language, or behavior areas, including but not limited to autistic spectrum disorder (ASD), down syndrome (DS), attention-deficit/hyperactivity disorder, cerebral palsy (CP), and learning disabilities [3]. These generally manifest during the developmental period, and their effects typically persist throughout life [3].

Historically speaking, developmental disabilities have been predominantly recognized for their negative impacts on the physical and psychosocial health of affected individuals [4]. Indeed, developmentally disabled children and their families can expect to encounter an endless series of daily challenges that can undermine the overall functioning of the entire family [5,6]. However, positive experiences have also been reported by parents raising children who have developmental disabilities, such as their feelings of appreciation, improved tolerance and sensitivity toward others, opportunities for learning, and deeper familial connections [7,8]. Effective advocacy, including promoting family-centered interventions and socioeconomic support, can reinforce well-being and resilience in such families [9,10].

Early intervention is an umbrella term encompassing a range of services and support that are provided for developmentally vulnerable infants, toddlers, and their families to enable them to achieve their optimal competencies and quality of life [11,12]. Robust neurological evidence supports the strong recommendation to provide early intervention in the first 3 years of life, which is a period of high brain plasticity [13-19]. This addresses 5 critical domains of development—the cognitive, physical, adaptive behavioral, social or emotional, and communication domains—to mitigate developmental challenges and maximize children’s developmental potential [13]. In addition, in that multidisciplinary and family-centered care (FCC) are critical principles for early intervention [14-18], the positive effects of early intervention extend to family outcomes including parents’ competency and appraisal, family relationships, and family quality of life [19-21].

In spite of substantial research that has demonstrated the positive effects of early interventions on children who have developmental disabilities and their families [4,22], several factors continue to impede the accessibility of such services, including complex referral and provision systems, long wait times, and geographical distances [23,24]. The COVID-19 pandemic has only exacerbated the challenges faced by these families, reducing their access to in-person services [25]. Rural areas and underdeveloped societies tend to be more vulnerable to these issues, aggravating existing health disparities [26,27]. In response to these challenges, telehealth has emerged as a solution that can help address the limitations of traditional early intervention methods, such as accessibility issues and resource shortages.

For several decades, telehealth has been extending its sphere of influence and making remarkable advancements [28]. Recently, the COVID-19 pandemic has accelerated its growth, going beyond a proper alternative to becoming an essential method of global health care [29]. While the terminology of telehealth is often used interchangeably with other terms, including telemedicine, telepractice, and teleintervention, telehealth is the broadest term that encompasses all types of remote health care services delivered via technology, as suggested in multiple definitions of telehealth [30-32]. World Health Organization defines it as any method of delivering “health care services, where patients and providers are separated by distance,” so long as they use “information and communication technologies for the exchange of information” [32]. Medicaid suggests a narrower definition of telehealth as permitting “2-way, real-time interactive communication between the patient and the physician or practitioner at the distant site” via audio or video components [30].

Previous research has investigated the effectiveness of telehealth across health conditions and health care settings [33,34]. Telehealth is a promising means of overcoming distance, contributing to the prevention of infections, and narrowing health disparities between urban and rural areas, as well as between developed and underdeveloped societies [28].

Young children who have developmental disabilities are among the populations that benefit from telehealth. Previous research has found that telehealth is as effective as or better than traditional methods of delivering rehabilitation, parent-mediated intervention, and parent coaching to children who have developmental disabilities [35-37]. A few reviews have demonstrated the success of early interventions via telehealth. Still, these include a wider range of ages, up to 7 years [38,39], despite the heterogeneity in children’s developmental stages. Further, these reviews tend to limit their focus to specific diagnoses (eg, ASD, CP) or particular types of intervention (eg, mobile apps, websites) [40,41]. To understand the implementation and effectiveness of early intervention via telehealth precisely, it is indispensable to focus on children aged 0-3 years and not to limit types of developmental disability or delay and telehealth modalities.

This systematic review compiled and synthesized the literature on early intervention via telehealth for children who have a high risk of developmental disabilities and their families, focusing on evaluating the implementation process and the effectiveness of these interventions. The main research question, structured in Patient, Interested Intervention, Comparison, Outcome (PICO) format, was “Is early intervention via telehealth (I) effective in improving outcomes (O) for infants and toddlers aged 0-3 years with or at high risk for developmental disabilities and their families (P), relative to conventional in-person intervention (C)?”

## Methods

### Methodology

The review was conducted and reported using systematic review methodologies from the Joanna Briggs Institute [42].

### Selection Criteria

The inclusion and exclusion criteria are described in Textbox 1. In this study, early intervention via telehealth was defined as using information and communication technologies to enable active interactions between children or families and service providers, either synchronously or asynchronously. This definition reflects the nature of early intervention, which involves active engagement and collaboration of families and providers [13]. Thus, studies adopting telehealth modalities only for recording, monitoring, self-management, assessment, or diagnosis were excluded, as they do not involve the interactive process integral to early intervention practices.

**Inclusion and exclusion criteria for study selection.**

**Inclusion criteria**

- Population: children aged 0-3 years with or at high risk of developmental disability
- Intervention: telehealth interventions involving interactions with a health care provider
- Outcomes: results including child or family outcomes
- Study design: empirical studies
- Year: January 2010 to April 2024
- Language: available in English

**Exclusion criteria**

- Population: children older than 3 years of age
- Intervention: adoption of telehealth modalities only for recording, monitoring, self-management, assessment, or diagnosis without interaction or feedback from health care providers
- Outcomes: results not reporting child outcomes
- Study design: nonempirical studies (eg, editorials, study protocols, or review studies)

### Search Strategy

A comprehensive literature search of 4 bibliographic databases (PubMed, Embase, CINAHL, and Web of Science) was conducted to identify studies using free keywords and the index vocabulary tailored for each database. The search strategy adopted was developed in collaboration with a librarian expert in the field. The search terms were a combination of three categories: (1) developmental disabilities, (2) telehealth, and (3) early intervention (Multimedia Appendix 1).

### Study Selection

For the study selection, all search references were imported into Covidence (Veritas Health Innovation), a management tool used to review studies. After the exclusion of duplicates, 2 reviewers independently screened the title and abstract, followed by a full-text review. Disagreements were resolved by the corresponding author. For data extraction, a template was developed using the software program Microsoft Excel to collate specific information from the included studies.

### Quality Assessment

Quality assessment was conducted using the mixed methods appraisal tool [43] to ensure the methodological quality of the studies included (Multimedia Appendix 2 [44-61]). This tool includes 5 distinct checklists for empirical studies across categories. Each criterion could be assigned to “yes,” “no,” or “can’t tell.” The total quality score for each article ranged from 0% to 100%, where higher scores indicated higher methodological quality. Two researchers independently appraised each study, and conflicts or criteria that were answered as “can’t tell” were discussed with the corresponding author. The threshold for inclusion was 60% or more.

### Data Extraction and Synthesis

Due to the heterogeneity of the populations, interventions, and outcome measures across studies, a narrative synthesis was conducted following the guidance of Popay et al [62]. Two researchers investigated the selected studies thoroughly to develop a standardized tabular form for the extraction and synthesis of the data. The synthesis included (1) the general characteristics of the studies that were included (authors, study location, study design, target functions, and technological modalities); (2) their population characteristics (diagnosis or condition, sample size, age, and sex); (3) the intervention characteristics (program title or theoretical base, provider or profession, description, control, and duration), and (4) the outcome characteristics (variables, measurements, and main findings). The frequency of the technological modalities and target functions across the included studies were described in independent matrix tables. Minor disagreements that arose during the process were resolved by consensus between the investigators. The corresponding author was brought in when consensus could not be reached.

## Results

### Overview

**Figure 1:**
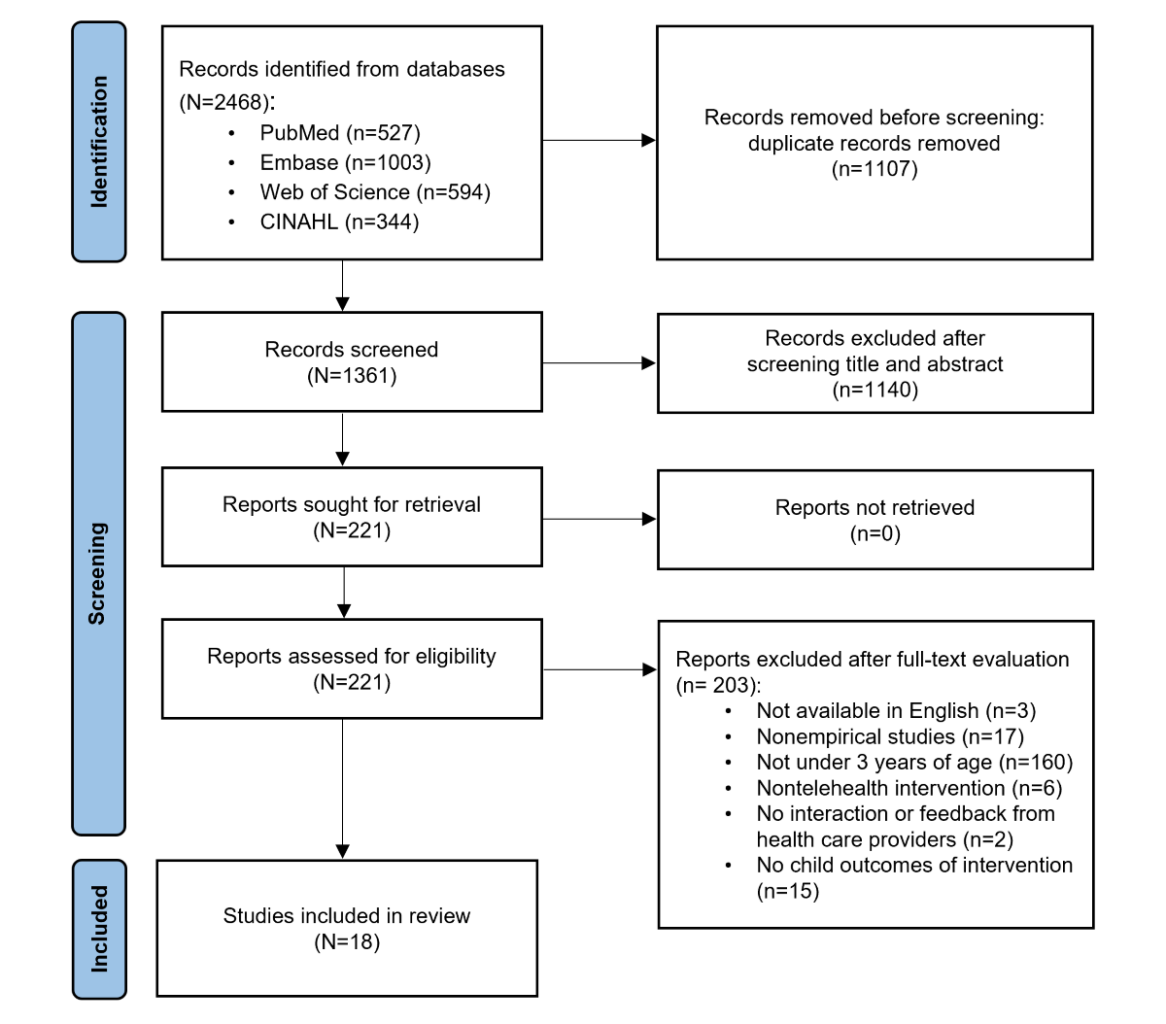
Flow diagram of study selection following the PRISMA (Preferred Reporting Items for Systematic Reviews and Meta-Analyses) guideline.

An electronic search yielded 2468 studies, of which 18 were ultimately included in the review. The screening process is detailed in the PRISMA (Preferred Reporting Items for Systematic Reviews and Meta-Analyses) diagram, shown in Figure 1.

### Quality Assessment

Of the 18 selected studies, 4 studies were evaluated using the checklist for quantitative randomized controlled trials, all scoring 100% (Multimedia Appendix 2). A checklist for quantitative non-randomized research was used to assess 13 studies. Of these, 7 studies scored 100%, 5 studies scored 80%, and 1 study scored 60%, due to an unclear indication of participant eligibility (n=3, 50%), an incomplete exhibition of outcome data (n=2, 33%), or an inadequate consideration of potential confounders (n=2, 33%). One study was categorized as a mixed methods study and scored 100% on the quality assessment.

#### General Characteristics of the Reviewed Studies

Of the 18 studies included, 13 were published between 2020 and 2024, following the onset of the COVID-19 pandemic (Multimedia Appendix 3 [44-61]). The studies were primarily conducted in the United States (n=10, 56%), with others from Canada (n=2, 11%), Brazil (n=3, 17%), Italy or Denmark (n=1, 6%), Iran (n=1, 6%), and Sweden (n=1, 6%). The designs of the studies were varied, including single-case designs (n=11, 61%), randomized controlled trials (n=4, 22%), and nonequivalent control group designs (n=3, 17%). The sizes of the samples ranged from single cases to 82 participants, including 8 studies featuring 6 or fewer participants.

#### Participant Characteristics

Most studies focused on children at risk for or diagnosed with ASD (n=10, 56%; Multimedia Appendix 3 [44-61]). Other studies included children at risk for CP (n=4, 22%), born preterm (n=1, 6%), with DS or developmental delays (n=1, 6%), with DS and visual impairment (n=1, 6%), or with a hearing impairment (n=1, 6%). Among the 12 that specified the sex of the child participant, 147 were boys (62.3%), and 89 were girls (37.7%). The ages of the children ranged from 5 to 37 months.

All studies, with the exception of one, which included a child and both of her parents [44], recruited participants as caregiver-child dyads. In all, 14 studies specified the caregiver’s sex; 170 (88.1%) were female, and 23 (11.9%) were male. Although none of the studies purposefully recruited mother-child dyads, seven ended up having 100% female caregivers, all of whom were the child’s mothers, except for 1 grandmother [45]. Eight studies reported the ages of the caregivers, which ranged from 20 to 48 years.

### Intervention Characteristics

#### Technological Modalities

Videoconferencing using devices such as smartphones, tablets, or personal computers was predominantly used (n=17, 94%) to deliver interventions (Table 1). The only exception was Sgandurra et al [46] who used an Internet of Things (IoT) baby gym, which enabled remote, highly personalized rehabilitation and monitoring. Websites or multimedia modules (n=2, 11%) and data sharing platforms or clouds (n=8, 44%) were used to facilitate data sharing between providers and families. Two studies used an IoT system or a wearable device to monitor children’s functioning [46,47].

**Table 1:** Frequencies of telehealth technological modalities used in the reviewed studies.

Author (year)	Videoconferencing	Website or module	Data sharing platform or cloud	IoT^a^ system or device
Akemoğlu et al (2022) [56]	✓	✓	✓	
Azzano et al (2023) [44]	✓	✓		
Bailey et al (2024) [48]	✓		✓	
Brian et al (2022) [45]	✓			
Daczewitz et al (2020) [49]	✓			
de Almeida Rodrigues et al (2023) [61]	✓			
Kunze et al (2021) [50]	✓		✓	
Lee et al (2023) [60]	✓		✓	
Lima et al (2023) [55]	✓			
Meadan et al (2016) [51]	✓		✓	
Pietruszewski et al (2020) [47]	✓			✓
Sadeghi et al (2022) [59]	✓			
Schlichting et al (2022) [52]	✓			
Sgandurra et al (2017) [46]				✓
Svensson et al (2024) [53]	✓			
Vismara et al (2013) [54]	✓		✓	
Vismara et al (2018) [58]	✓		✓	
Vismara et al (2012) [57]	✓		✓	

^a^IoT: Internet of Things.

#### Intervention Contents

The intervention characteristics are given in Multimedia Appendix 4 [44-61]. Among the 17 studies that used videoconferencing, all but 1 study [45], which included both individual and group sessions, conducted one-on-one sessions to provide parent training, coaching, or both. In the sessions, the providers delivered knowledge of strategy, answered questions, monitored caregiver-child interactions, and provided real-time feedback.

Nine out of 17 studies had children participate in live sessions to observe caregivers’ strategy implementation, monitor their progress, and provide coaching at the moment [44,47-54]. The remaining 8 studies involved only researchers and caregivers, focusing on the delivery of strategies so that caregivers could apply them to their children later. Four studies prescribed dosage for caregivers’ self-practice outside of the videoconferencing sessions [54,55,57, 60], where 3 required caregivers to log their progress, whether online or offline. In addition, 7 studies individually tailored the goals and plans of the programs as a result of discussions conducted between providers and caregivers, which increased the extent of the individualization of the interventions offered [49,51,54,55,57,58,61].

In addition to videoconferencing, in 5 studies, the caregivers [53,54,56-58] received training in multimedia modules provided through web platforms or DVDs, primarily containing video and written instructions. The caregivers were instructed to use the modules for self-teaching in a predetermined sequence or at their own pace. The IoT baby gym system that was used by Sgandurra et al [46] provided daily goal-directed activities based on the child’s condition monitored through video cameras, wearable sensors, and other sensorized components, such as mats and toys.

The interventions for all 18 studies were provided by researchers who had relevant experience with the target population. Nine studies indicated the professions or professional qualifications of service providers, which included special educators (n=3, 17%), physiotherapists/occupational therapists (n=3), behavior analysts (n=2, 11%), and psychologists (n=1, 6%). The average intervention was approximately 10.2 weeks long, ranging from 4 to 29 weeks, except for 1 study, which only stated the number of sessions (42-57 sessions) [48]. The average frequency was approximately 1.6 sessions per week, with the 4 aforementioned studies including additional self-practice components [52,53,55,60]. Each session continued for an average of approximately 54.3 minutes, with a range of 15 to 90 minutes.

#### Effectiveness of Interventions

All of the included studies suggested the overall effectiveness of the interventions on child outcomes, with some degree of variability (Multimedia Appendices 3 and 4 [44-61]). In each of the 18 studies, the interventions targeted one or more domains of child development (Table 2). Among the 5 early-intervention domains, the development of communication was most frequently addressed (n=9, 50%), as measured through variables such as communicative behaviors (eg, initiation, response, and vocal imitation) and the number of words used. Studies targeting social/emotional development (n=6, 33%) assessed social behaviors, including joint attention, social turn-taking, and behavior imitation. General or specific motor skills were measured for physical development (n=6, 33%). The development of adaptive behavior (n=4, 22%) was evaluated with flexible and inflexible behavior, repetitive and restricted behaviors, instrumental turn-taking, and daily activities. It should be noted that cognitive development was not specifically targeted as part of any of the included studies.

**Table 2:** Frequencies of child functions targeted in the reviewed studies based on early intervention domains [13].

Author (year)	Target function domain				
	Cognitive (n=0)	Physical (n=6)	Adaptive behavior (n=4)	Social or emotional (n=6)	Communication (n=9)
Akemoğlu et al (2022) [56]					✓
Azzano et al (2023) [44]				✓	✓
Bailey et al (2024) [48]					✓
Brian et al (2022) [45]					✓
Daczewitz et al (2020) [49]					✓
de Almeida Rodrigues et al (2023) [61]		✓	✓		
Kunze et al (2021) [50]			✓		
Lee et al (2023) [60]			✓	✓	
Lima et al (2023) [55]		✓		✓	
Meadan et al (2016) [51]					✓
Pietruszewski et al (2020) [47]		✓			
Sadeghi et al (2022) [59]			✓		
Schlichting et al (2022) [52]		✓			
Sgandurra et al (2017) [46]		✓			
Svensson et al (2024) [53]		✓			
Vismara et al (2012) [57]				✓	✓
Vismara et al (2013) [54]				✓	✓
Vismara et al (2018) [58]				✓	✓

More particularly, among the child outcomes, the communication skills of children with or at risk for ASD were the most frequently reported (n=7, 39%), followed by the motor function of infants having or at biological risk for CP (n=4, 22%). The adaptive behavior of children with or at risk for ASD has been assessed in 3 studies [50,59,60]. Six studies included control groups, three of which provided in-person or traditional care to the control groups. Two studies reported nonsignificant differences between the invention (telehealth) and the control (in-person or traditional care) groups.

The outcomes for caregivers and families were reported in 12 studies, among which 10 demonstrated increased or appropriate levels of caregivers’ implementation fidelity for learned strategies. Implementation-related outcomes, such as acceptability, feasibility, satisfaction, and cost efficiency were also measured. In addition, interventions were reported to improve clinical outcomes for caregivers and families, including parenting stress (n=2, 11%), caregiver engagement (n=1, 6%), self-efficacy (n=1, 6%), and home environment (n=1, 6%), although these did not consistently reach statistical significance.

## Discussion

### Principal Findings

This review synthesized the existing literature on early interventions through telehealth for young children who have developmental risks and examined their implementation and effectiveness. A distinguishing feature of this review is that the child participants were restricted to the ages of 0-3 years, strictly following the extensively recommended criteria for early intervention. Thereby, this study enabled a concrete inspection of current practices in telehealth interventions that were precisely aimed at young children during their first 3 years and their impact.

### Effectiveness

This review identified the overall effectiveness of early interventions via telehealth for both children and their families. In particular, children showed improvement in their targeted abilities and behaviors, in general, corresponding to the caregivers’ enhanced or sufficient fidelity for implementing learned strategies. The effectiveness was comparable to those of in-person approaches. This finding is consistent with previous studies that have addressed the effectiveness of telehealth interventions for children and youths with developmental disabilities [35]. McCarthy et al [63] and Bharat et al [40] suggested a potential for telehealth or mobile apps in delivering early intervention for children aged under 6 with ASD or hearing difficulties. For older children and adolescents, multiple reviews have indicated that telehealth interventions for ASD [41,64,65] and CP [66] are at least as effective as traditional methods. Telehealth interventions demonstrate sustained effectiveness throughout childhood in children who have developmental challenges.

#### Scope of Child Conditions and Target Outcomes

However, early interventions through telehealth included in the studies were less comprehensive, with limited populations or target functions relative to traditional in-person interventions. Most interventions targeted either communication development in children who have ASD during their second or third years of life or motor development in infants with CP; none of the included studies targeted or examined child outcomes in relation to the cognitive domain. Cognitive ability serves as the foundation for early childhood development, and it affects overall health and socioeconomic status during the later stages of life [67,68]. This underscores the importance of early intervention for enhancing cognitive development [69,70], highlighting further attention to this omission. The alienation of cognitive outcomes is also observed in telehealth interventions for children who have developmental disabilities and are aged more than 3 years [41,65,71]. This suggests that adopting telehealth in comprehensive interventions including the cognitive domain for children having developmental disabilities or delays could accompany difficulties. Some conditions require specialized skills that are difficult to deliver virtually [72], and certain assessments require long periods or physical materials that may not be feasible using telehealth [73,74].

Despite these challenges, a comprehensive approach is uncompromisable for early intervention for children with developmental disabilities [75,76]. As a result of the interrelated nature of all aspects of development, such that the growth or deficiency in 1 area influences that of others, thereby either promoting or hindering overall development [13]. Thus, the study of early intervention for young children with developmental challenges has consistently striven to encompass cognitive, physical, adaptive behavior, social or emotional, and communication domains, and it has even expanded to include sensory integration, nutrition, and sleep [75,77-80]. To establish telehealth as a viable method for the delivery of early intervention, future research is encouraged to cover a more comprehensive range of developmental challenges.

#### Telehealth Modalities

The most widely used form of early intervention through telehealth was synchronous caregiver training or coaching through videoconferencing, engaging caregivers as the main implementers. The caregivers generally reported satisfaction with telehealth care. Real-time videoconferencing sessions between providers and caregivers produced positive feedback, in particular when it was combined with multimedia modules.

Telehealth interventions commonly meet certain barriers, including technological issues, lack of digital literacy, and negative perceptions of telehealth [81,82]. In pediatric care, telehealth assigns extra responsibility to caregivers for delivering interventions and maintaining an optimal environment for remote communication, which some find burdensome [83,84]. The caregivers in the included studies reported occasional internet issues and discomfort in moderating the home setting; however, these did not critically reduce their satisfaction with telehealth modalities.

Consistent reports of decent social validity could be attributed to the participants’ ages and locations. Youths, internet access, and technological competency facilitate the use of telehealth, while old age, technological disconnection, and insufficient understanding of telehealth pose barriers [81,85]. Studies of early-intervention studies typically involve young individuals who have high digital literacy [86] and are often conducted in environments having stable internet connections, which likely contributes to positive feedback on telehealth. Future research should account for the digital divide according to age and region, as it prevents the generalizability of the results.

### Family-Centered Care

The concept of early intervention is grounded in the principle of FCC, as has been established in key definitions and purposes emphasizing enhancements to parental capacity and supporting parent-mediated interventions [11,13]. The integration of telehealth into early intervention strengthens home-based family participation and caregiver competence [87-89], promoting active family engagement within a natural environment, and leading to favorable outcomes for both child and family [39].

While the included studies incorporated FCC, there is potential for further improvement in its application in early intervention, in particular through telehealth [90]. Dyad-recruiting and caregiver coaching/training, the most common strategies used by the included studies, are reported to be effective in child development, caregiver implementation, and child-caregiver interaction [13,22,91-93]. However, a notable sex imbalance was seen in caregivers who were participating in the studies, with mothers predominantly participating. This inclination toward the mother aligns with prior FCC research, which attributes it to traditional sex roles, lack of tailored services, and limited awareness of both families and providers [22,94-97]. Nonetheless, fathers’ engagement is linked to the improved cognitive, emotional, behavioral, and psychopathological development of young children [98-100], and involving siblings as playmates or models is associated with enhanced peer relationships and social skills [101,102]. Future research should explore strategies to increase father and sibling involvement in FCC interventions.

In addition, most included studies had family outcomes that were limited to caregivers’ implementation in the absence of clinical outcomes, underscoring the need for further exploration of caregiver and family outcomes to develop and evaluate FCC-based early interventions through telehealth. Research indicates that parents’ physical and mental health directly affects their ability to provide care and support for their children, which, in turn, impacts children’s developmental progress and overall well-being [103,104]. By contrast, the severity of children’s developmental disabilities can also affect parental health, producing an interplay within families [105-107]. Therefore, more comprehensive research is required to explore the whole family’s outcomes when evaluating the effects of telehealth early interventions.

### Multidisciplinary Approach

A multidisciplinary approach should also be considered, as each intervention in the included studies was conducted by individuals from a few professions, including physiotherapists, behavior analysts, and special educators. While the focused expertise of the providers could allow for intensive interventions in particular areas, collaboration among diverse professions fosters a comprehensive understanding of conditions, integrative problem-solving, and effective risk management, ultimately enhancing the quality and outcomes of the intervention [108-110]. A multidisciplinary team, including education, medical care, rehabilitation, community service, and family, can reflect the holistic nature of children who have developmental disabilities and their families [111,112]. In particular, thanks to early intervention, a multidisciplinary approach can be posited as an essential principle for the promotion of individualization and family engagement and achieving optimal development [113].

### Limitations

To the best of our knowledge, this is the first systematic review to synthesize the use of telehealth in early intervention for young children aged 0-3 years and their families, involving developmental challenges. However, this study has some limitations.

First, a large portion of the included studies used single-case designs, which pose challenges in generalizing findings to broader populations. This limitation hindered our analysis to compare the overall effectiveness of telehealth interventions to traditional methods or evaluate variability in effectiveness across different groups based on factors such as participants’ age, sex, and developmental disabilities of the children.

Consequently, we were unable to calculate effect sizes for a statistical synthesis, limiting our ability to draw definitive conclusions about the effectiveness of early intervention delivered through telehealth.

While our search terms were meticulously developed in collaboration with an institutional librarian specializing in medical research, the scope of our search could be further refined. The exclusion of gray literature may have omitted informal data or professional experiences beyond traditional research settings. Additionally, limiting the review to published studies introduces a potential risk of publication bias, as studies with positive results are more likely to be published. Future research incorporating a wider range of integrative works, including gray literature, could enhance the generalizability and provide a more comprehensive understanding of telehealth’s impact on early intervention.

Finally, the findings predominantly come from developed, primarily Western countries. This limitation may be due to restricting the search to English-language studies, as well as the concentration of related research in these regions. In many low- and middle-income countries, early intervention remains a lesser-known concept [114], and the necessary infrastructure for telehealth delivery is often lacking [115]. These considerations may explain the geographic biases of this review; nonetheless, the findings serve as a foundation for guiding future research and policies to expand access to early intervention via telehealth in underserved communities.

### Conclusions

This review highlights the potential that telehealth has for early intervention for young children having developmental challenges and their families. By synthesizing the existing literature, we observed that telehealth, mainly in the form of synchronous caregiver training or coaching, can effectively enhance child and family outcomes across various areas. Despite its limitations, the adoption of telehealth technologies in early intervention is a promising direction for researchers, clinicians, and educators to deliver holistic, FCC for young children with developmental disabilities at a distance. This integrative approach will ultimately benefit children who have developmental challenges and their families in remote or underserved areas by improving developmental outcomes and the quality of life within families. Finally, we call for additional high-quality research that expands the scope of telehealth early interventions and ensures a comprehensive, multidisciplinary approach for children with developmental disabilities and their families, which can generate robust and generalizable evidence.

## Appendix

Multimedia Appendix 1 Detailed search terms.

Multimedia Appendix 2 The quality assessment based on the MMAT (Mixed Methods Appraisal Tool) tool.

Multimedia Appendix 3 Summary of main characteristics of the included studies’ locations, designs, participants, primary technological modalities, and main findings.

Multimedia Appendix 4 Characteristics of early interventions via telehealth and measurements for children aged 0-3 years in the reviewed studies.

Multimedia Appendix 5 PRISMA 2020 checklist.

## Abbreviations

ASD: autistic spectrum disorder
CP: cerebral palsy
DS: down syndrome
FCC: family-centered care
IoT: Internet of Things
PICO: Patient, Interested Intervention, Comparison, Outcome
PRISMA: Preferred Reporting Items for Systematic Reviews and Meta-Analyses

## Data Availability

All data generated or analyzed during this study are included in this published article and its supplementary information files.
