# Supplementary material for: Early Intervention for Children With Developmental Disabilities and Their Families via Telehealth: Systematic Review
Source: J Med Internet Res. 2025 Jan 17;27:e66442. doi: 10.2196/66442 (PMC11786141; doi:10.2196/66442)
Supplement: Multimedia Appendix 1 [file jmir_v27i1e66442_app1.docx]

**Table S1. Detailed search terms**

| Database | Search terms |
| --- | --- |
| PubMed | ("child development disorders, pervasive"[Mesh] OR "developmental disabilities"[Mesh] OR "neurodevelopmental disorders"[Mesh] OR "Autism Spectrum Disorder"[Mesh] OR "intellectual disability"[Mesh] OR "down syndrome"[Mesh] OR "cerebral palsy"[Mesh] OR "developmental disabilit*"[Title/abstract] OR "developmental disorder"[Title/abstract] OR "developmental delay"[Title/abstract] OR Autism[Title/abstract] OR Autistic[Title/abstract] OR ASD[Title/abstract] OR "Down syndrome"[Title/abstract] OR "Brain Damage, Chronic"[Mesh] OR "cerebral palsy"[Title/abstract] OR "brain damage*"[Title/abstract] OR "brain injur*"[Title/abstract]) AND ("digital health"[Mesh] OR videoconferencing[Mesh] OR telerehabilitation[Mesh] OR telenursing[Mesh] OR videoconferenc*[Title/abstract] OR "video conferenc*"[Title/abstract] OR telerehabilitation[Title/abstract] OR tele-rehabilitation[Title/abstract] OR telenursing[Title/abstract] OR telehealth[Title/abstract] OR telemedicine[Title/abstract] OR telepractice[Title/abstract] OR telecare[Title/abstract] OR "virtual delivery"[Title/abstract]) AND (child[Mesh] OR infant[Mesh] OR child*[Title/abstract] OR infant*[Title/abstract] OR toddler*[Title/abstract]) |
| Embase | ('developmental disorder'/exp OR 'developmental delay'/exp OR 'pervasive developmental disorder not otherwise specified'/exp OR autism/exp OR 'down syndrome'/exp OR 'cerebral palsy'/exp OR 'developmental disabilit*':ti,ab,kw OR 'developmental disorder':ti,ab,kw OR 'developmental delay':ti,ab,kw OR autism:ti,ab,kw OR autistic:ti,ab,kw OR ASD:ti,ab,kw OR 'down syndrome':ti,ab,kw OR 'cerebral palsy':ti,ab,kw OR 'brain damage*':ti,ab,kw OR 'brain injur*':ti,ab,kw) AND ('digital health'/exp OR telehealth/exp OR telecare/exp OR telenursing/exp OR telemedicine/exp OR videoconferencing/exp OR videoconferenc*:ti,ab,kw OR 'video conferenc*':ti,ab,kw OR telerehabilitation:ti,ab,kw OR tele-rehabilitation:ti,ab,kw OR telenursing:ti,ab,kw OR telehealth:ti,ab,kw OR telemedicine:ti,ab,kw OR telepractice:ti,ab,kw OR telecare:ti,ab,kw OR 'virtual delivery':ti,ab,kw) AND (child/exp OR toddler/exp OR infant/exp OR child*:ti,ab,kw OR toddler*:ti,ab,kw OR infant*:ti,ab,kw) |
| CINAHL | (MH child development disorders, pervasive OR MH developmental disabilities OR MH autistic disorder OR MH down syndrome OR MH intellectual disability OR (TI developmental disabilit* or AB developmental disabilit*) OR (TI developmental disorder or AB developmental disorder*) OR (TI developmental delay or AB developmental delay) OR (TI autis* or AB autis*) OR (TI ASD or AB ASD) OR (TI down syndrome or AB down syndrome) OR MH brain damage, chronic OR MH cerebral palsy OR (TI brain damage or AB brain damage) OR (TI brain injur* or AB brain injur*) OR (TI cerebral palsy or AB cerebral palsy)) AND (MH telehealth OR MH telenursing OR MH telerehabilitation OR MH videoconferencing OR MH digital health OR (TI telehealth or AB telehealth) OR (TI telerehabilitation or AB telerehabilitation) OR (TI tele-rehabilitation or AB tele-rehabilitation) OR (TI telepractice or AB telepractice) OR (TI telemedicine or AB telemedicine) OR (TI telenurinsg or AB telenursing) OR (TI telecare or AB telecare) OR (TI videoconferencing or AB videoconferencing) OR (TI video conferencing or AB video conferencing) OR (TI virtual delivery or AB virtual delivery)) AND (MH child, preschool OR MH children with disabilities OR MH infant OR (TI child or AB child) OR (TI toddler or AB toddler) OR (TI infant or AB infant)) |
| Web of Science | ((TI=(“developmental disorder*” OR “developmental disabilit*” OR “developmental delay*” OR “intellectual disabilit*” OR autistic OR autism OR ASD OR “down syndrome” OR “brain damage*” OR “brain injury” OR “cerebral palsy”) OR AB=(“developmental disorder*” OR “developmental disabilit*” OR “developmental delay*” OR “intellectual disabilit*” OR autistic OR autism OR ASD OR “down syndrome” OR “brain damage*” OR “brain injury” OR “cerebral palsy”)) AND (AK=(“developmental disorder*” OR “developmental disabilit*” OR “developmental delay*” OR “intellectual disabilit*” OR autistic OR autism OR ASD OR “down syndrome” OR “brain damage*” OR “brain injury” OR “cerebral palsy”)) AND (TI=(child* OR toddler* OR infant*) OR AB=(child* OR toddler* OR infant*) OR AK=(child* OR toddler* OR infant*))  AND (TI=(videoconferenc* OR “video conferenc*” OR telerehabilitation OR telenursing OR telehealth OR telemedicine OR telepractice OR telecare OR “virtual delivery”) OR AB=(videoconferenc* OR “video conferenc*” OR telerehabilitation OR telenursing OR telehealth OR telemedicine OR telepractice OR telecare OR “virtual delivery”) OR AK=(videoconferenc* OR “video conferenc*” OR telerehabilitation OR telenursing OR telehealth OR telemedicine OR telepractice OR telecare OR “virtual delivery”))) AND PY=(2024 OR 2023 OR 2022 OR 2021 OR 2020 OR 2019 OR 2018 OR 2017 OR 2016 OR 2015 OR 2014 OR 2013 OR 2012 OR 2011 OR 2010) |
