# Supplementary material for: Early Intervention for Children With Developmental Disabilities and Their Families via Telehealth: Systematic Review
Source: J Med Internet Res. 2025 Jan 17;27:e66442. doi: 10.2196/66442 (PMC11786141; doi:10.2196/66442)
Supplement: Multimedia Appendix 2 [file jmir_v27i1e66442_app2.docx]

**Table S2.** Appraisal of the methodological quality of included studies from the MMAT form

| Quantitative randomized controlled trials | | | | | | |
| --- | --- | --- | --- | --- | --- | --- |
|  | Appropriate randomization | Groups comparable at baseline | Complete outcome data | Blinded outcome assessors | Adherence to intervention | Total (%) |
| Lima et al. (2023) | Y | Y | Y | Y | Y | 100 |
| Pietruszewski et al. (2020) | Y | Y | Y | Y | Y | 100 |
| Sgandurra et al. (2017) | Y | Y | Y | Y | Y | 100 |
| Vismara et al. (2016) | Y | Y | Y | Y | Y | 100 |
| Quantitative non-randomized research | | | | | | |
|  | Representation of the target population | Appropriate measurements | Complete outcome data | Confounders accounted for | Interventions administered as intended | Total (%) |
| Akemoğlu et al. (2022) | N | Y | Y | Y | Y | 80 |
| Azzano et al. (2023) | N | Y | Y | Y | Y | 80 |
| Bailey et al. (2024) | Y | Y | Y | Y | Y | 100 |
| Brian et al. (2022) | Y | Y | Y | Y | Y | 100 |
| Daczewitz et al. (2020) | Y | Y | Y | Y | Y | 100 |
| de Almeida Rodrigues et al. (2023) | Y | Y | Y | Y | Y | 100 |
| Kunze et al. (2021) | Y | Y | Y | N | Y | 80 |
| Meadan et al. (2016) | Y | Y | Y | Y | Y | 100 |
| Sadeghi et al. (2022) | Y | Y | N | Y | Y | 80 |
| T. Schlichting et al. (2022) | Y | Y | Y | N | Y | 80 |
| Svensson et al. (2024) | Y | Y | Y | Y | Y | 100 |
| Vismara et al. (2012) | N | Y | N | Y | Y | 60 |
| Vismara et al. (2013) | Y | Y | Y | Y | Y | 100 |
| Mixed-method | | | | | | |
|  | Adequate rationale of using mixed methods design | Effective integration of study components | Adequate interpretation of integrated outputs | Addressing of inconsistencies between results | Adherence to quality criteria of each method | Total (%) |
| Lee et al. (2023) | Y | Y | Y | Y | Y | 100 |
