# Supplementary material for: Early Intervention for Children With Developmental Disabilities and Their Families via Telehealth: Systematic Review
Source: J Med Internet Res. 2025 Jan 17;27:e66442. doi: 10.2196/66442 (PMC11786141; doi:10.2196/66442)
Supplement: Multimedia Appendix 3 [file jmir_v27i1e66442_app3.docx]

**Table S3.** Summary of main characteristics of the included studies’ locations, designs, participants, primary technological modalities, and main findings.

| Author(s) (year) | Study location | Study design | Diagnosis/ condition | Sample size | Child   - Age (months) - Sex | Caregiver   - Age (years) - Sex | Technology | Main findings |
| --- | --- | --- | --- | --- | --- | --- | --- | --- |
| **ASD or at risk of ASD** | | | | | | | | |
| Azzano et al. (2023) | Canada | Single-case design (pre-post) | at risk of ASD | 1 | - 30 - Male | - 25 (mother), 35 (father) - M: 1, F: 1 | Video conferencing | The intervention increased caregiver fidelity and improved the child’s target behaviors. |
| Bailey et al. (2024) | USA | Single-case design (pre-post) | ASD | 3 | - 27, 31, 35 - M: 2, F: 1 | - 20, 43, 48 - M: 0, F: 3 | Video conferencing | The intervention increased caregiver fidelity, and outcomes in child communication skills varied among participants. |
| Brian et al. (2022) | Canada | Nonequivalent control group design (pre-post) | ASD or clinical impression | 82 (IG 37, CG 45) | - Mean: 30.49 - M: 65, F: 17 | - N/S - M: 12, F: 70 | Video conferencing | Both interventions significantly increased caregiver fidelity and self-efficacy, with no significant differences between groups. In-person intervention was significantly more effective in reducing parenting stress.  Both interventions significantly improved children’s communication skills and ASD symptoms, with no significant differences between groups. |
| Kunze et al. (2021) | USA | Single-case design (pre-post) | ASD or in waitlist | 6 | - Mean (SD): 30.83 (4.77) - M: 2, F: 4 | - Mean (SD): 34.5 (8.01) - M: 0, F: 6 | Video conferencing | The intervention increased caregiver fidelity, enhancing the children’s adaptive behaviors and ASD symptoms. |
| Lee et al. (2023) | USA | Single-case design (pre-post) | ASD | 1 | - 28 - Male | - N/S - Female | Video conferencing | The intervention increased the child’s social communication skills overall, with the extent of improvement varying across measures. |
| Meadan et al. (2016) | USA | Single-case design (pre-post) | ASD | 3 | - 2, 2, 3 years - N/S | - N/S - M: 0, F: 3 | Video conferencing | The virtual intervention enhanced the caregiver fidelity in both quantity and quality, contributing to the improvements of the children’s communication behaviors. |
| Sadeghi et al. (2022) | Iran | Nonequivalent control group design (pre-post) | at risk of ASD + excessive screentime | 40 (IG 20, CG 20) | - Mean (SD): IG 30.45 (5.83), CG 31.90 (7.90) - N/S | - Mean (SD): IG 31.45 (3.58), CG 32.4 (5.83) - M: 0, F: 40 | Video conferencing | Both virtual and in-person interventions significantly improved the children’s adaptive behaviors and autism symptoms, with no significant differences.  Both interventions significantly reduced caregivers’ parenting stress, with no significant differences. |
| Vismara et al. (2012) | USA | Single-case design (pre-post) | ASD | 9 | - Mean (SD): 28.89 (7.64) - N/S | - N/S - M: 2, F: 7 | Video conferencing DVD module | The intervention significantly improved caregiver fidelity and child social communication behaviors. |
| Vismara et al. (2013) | USA | Single-case design (pre-post) | ASD | 9 | - Mean (SD): 27.5 (9.24) - N/S | - N/S - M: 1, F: 7 | Video conferencing Online module | The intervention enhanced caregiver fidelity and engagement, with a positive correlation between the two.  The intervention improved children’s social communication behavior overall, with variability across sessions. |
| Vismara et al. (2018) | USA | RCT | ASD | 24 (IG 14, CG 10) | - Mean (SD): IG 31.9 (10.4), CG 27.2 (7.9) - M: 17, F: 7 | - N/S - M: 5, F: 19 | Video conferencing Online module | The intervention enabled more caregivers to achieve proper fidelity relative to the control.  For both interventions, children’s social communication behavior showed significant improvements, with no significant difference. |
| **CP or at risk of CP** | | | | | | | | |
| Lima et al. (2023) | Brazil | RCT | at risk of CP | 28 (IG 14, CG 14) | - Mean (SD): IG 6.34 (2.15), CG 6.03 (2.15) - M: 15, F: 13 | - Mean (SD): IG 34.53 (5.46), CG 31.85 (4.89) - N/S | Phone or video call | The virtual intervention showed significantly greater effects on children’s motor skills and participation compared to the in-person intervention, while the home environment did not significantly change in either group. |
| Pietruszewski et al. (2020) | USA | Pilot RCT | CP | 13 (IG 7, CG 6) | - Median (range): IG 12 (10-15), CG 10 (9-11) - N/S | - N/S - N/S | Video conferencing  IoT device | The virtual intervention significantly improved the children’s unimanual fine motor skills compared to the waitlist control. |
| Schlichting et al. (2022) | Brazil | Single-case design (pre-post) | at risk of CP | 10 | - Mean (SD): 6.2 (3.78) - M: 4, F: 6 | - Mean (SD): 30 (SD 5.31) - M: 0, F: 10 | Video conferencing | The intervention improved the children’s motor skills. |
| Svensson et al. (2024) | Sweden | Nonequivalent control group design (pre-post) | at risk of CP | 38 (IG 20, CG 18) | - Mean (SD): IG 7 (2.8), CG 6 (1.7) - M: 20, F: 18 | - N/S - N/S | Video conferencing Web platform | Both virtual and in-person interventions improved children’s motor skills of the affected hand, with no significant differences. |
| **Other conditions** | | | | | | | | |
| Akemoğlu et al. (2022) | USA | Single-case design (pre-post) | DS or developmental delay | 3 | - 34, 36, 37 - N/S | - 36-45 - M: 1, F: 2 | Online module  Video conferencing | The intervention increased caregiver fidelity, which contributed to improvements in the children’s communication behaviors. |
| Daczewitz et al. (2020) | USA | Single-case design (pre-post) | DHH | 1 | - 26 - Female | - 32 - Male | Video conferencing | The intervention enhanced the quality of caregiver implementation with variability, contributing to some improvements in the child’s communication behaviors. |
| de Almeida Rodrigues et al. (2023) | Brazil | Single-case design (pre-post) | DS and visual impairment | 1 | - 5 - Male | - 39 - Female | Video conferencing | The intervention improved the child’s motor skills and daily activities, helping with the substantial achievement of individualized goals. |
| Sgandurra et al. (2017) | Italy/ Denmark | RCT | preterm infants | 41 (IG 19, CG 22) | - Mean (SD): IG 3.6 (0.4), CG 4.1 (1.0) - M: 19, F: 22 | - N/S - N/S | IoT system | The intervention significantly improved motor skills and visual capacity compared to the standard care control. |

ASD: Autism Spectrum Disorder; CG: control group; CP: cerebral palsy; DHH: deaf or hard-of-hearing; DS: Down Syndrome; IG: intervention group
