# Supplementary material for: Early Intervention for Children With Developmental Disabilities and Their Families via Telehealth: Systematic Review
Source: J Med Internet Res. 2025 Jan 17;27:e66442. doi: 10.2196/66442 (PMC11786141; doi:10.2196/66442)
Supplement: Multimedia Appendix 4 [file jmir_v27i1e66442_app4.docx]

**Table S4.** Characteristics of early interventions via telehealth and measurements for children aged 0-3 years in the reviewed studies.

| Author(s) (year) | “Program”/ Theoretical base | Provider/ Professional | Intervention Description | Control intervention | Duration   - Dosage: timespan | Variables | Measurements |
| --- | --- | --- | --- | --- | --- | --- | --- |
| **ASD or at risk of ASD** | | | | | | | |
| Azzano et al. (2023) | “Parent Intervention for Children at Risk for Autism (PICARA)”/  BST | Researchers (N/S) | VC-based training/coaching with parents practicing discrete trial training strategies with each other and with their child, receiving instant feedback | N/A | 29 weeks   - Once/week: 60 min/session | Child:  Vocal & nonvocal manding  Motor imitation with objects  Vocal imitation  Motor imitation with body | Poems |
|  |  |  |  |  |  | Implementation:  Fidelity, satisfaction | Checklist, questionnaire |
| Bailey et al. (2024) | NDBI (EMT) | BCBA, doctoral student in early childhood special education | Alternating VC-based individual caregiver training and child-involved coaching | N/A | N/S   - 42–57 sessions | Child:  Number of different words | Video transcription, CDI |
|  |  |  |  |  |  | Implementation:  Fidelity, acceptability, feasibility | Checklist, video coding, surveys, semi-structured interview |
| Brian et al. (2022) | "Virtual Group-Based Social ABCs”/  ABA | Coaches, facilitators (N/S) | Alternating VC-based individual coaching and group training (3–5 families): content explanation and participant discussion | Same intervention in-person | 6 weeks   - 6 group VC sessions: 90min/session - 3 individual VC sessions: 60 min/session | Child:  Vocal responsivity, word use & comprehension, ASD symptoms | Video coding, CDI, APSI |
|  |  |  |  |  |  | Caregiver/family:  Parenting stress, parent self-efficacy | PSI-SF, parent self-efficacy questionnaire |
|  |  |  |  |  |  | Implementation:  Fidelity, acceptability | questionnaire |
| Kunze et al. (2021) | ABA | Behavior analyst | VC-based parent-training with content explanation  VC-based parent-coaching with child: observation of parent-child interaction, subsequent feedback | N/A | 7-8 weeks   - 14–16 VC sessions: 40 min/session | Child:  Flexible & inflexible behavior, RRBIs, risk for ASD | Video observation, RBS-EC, M-CHAT-R/F |
|  |  |  |  |  |  | Implementation:  Fidelity | Video observation |
| Lee et al. (2023) | “The Social Turn-Taking Intervention”/  PML | Early childhood educator | VC-based parent-training: content explanation, discussion, review of recorded practice  Guided daily practice with logbook recording | N/A | 12 weeks   - 12 sessions: 60 min/session - Self-practice (daily): 30 min/day | Child:  Social turn-taking, instrumental turn-taking, focusing on face, initiating joint attention, responding to joint attention | Video coding |
|  |  |  |  |  |  | Implementation:  Fidelity | Checklist |
| Meadan et al. (2016) | “Internet-Based Parent-Implemented Communication Strategies (iPiCS)”/  Naturalistic teaching | Special educator | Preliminary VC-based parent-training: content explanation, individual planning before coaching sessions  VC-based parent-coaching with child: observation of parent-child interaction, feedback, discussion | N/A | 14 weeks   - 1 training session: 45 min - 27 VC coaching sessions: 30 min/session | Child:  Communicative behavior (responding, initiating) | Video coding |
|  |  |  |  |  |  | Implementation:  Fidelity | Video coding |
| Sadeghi et al. (2022) |  | Child clinical psychologist | VC-based group parent-training (4 caregivers) with education  VC-based individual parent-coaching: review of past practice, discussion, consulting | Same intervention in-person | 7 weeks   - 4 group VC sessions: 90 min/session - 3 individual VC sessions: 30 min/session | Child:  Repetitive behaviors, ASD symptoms | RBS-R, GARS-2 |
|  |  |  |  |  |  | Caregiver/family:  Parenting stress | PSI-SF |
| Vismara et al. (2012) | ABA (ESDM) | Therapists (N/S) | VC-based parent-training/coaching: review of past practice, discussion, education, individual goal setting/planning  Self-directed module-based training via DVD: educational materials, preset order instructions for upcoming sessions | N/A | 12 weeks   - 12 VC sessions: 60 min/session - Modules: 20 min/module | Child:  Social communication behavior, attention, initiation | Video transcription, CBRS |
|  |  |  |  |  |  | Implementation:  Feasibility, acceptability, fidelity | Questionnaire, ESDM fidelity score, MBRS |
| Vismara et al. (2013) | ABA (ESDM) | Therapists trained for the P-ESDM model | VC-based parent-training/coaching with child: review of past practice, observation of parent-child interaction, discussion, individual planning  Self-directed module-based training via web platform: education (text/video), additional resources, caregiver-therapist communication | N/A | 12 weeks   - 12 VC sessions: 90 min/session - Modules: self-paced | Child:  Response, communicative development | Video transcription, CDI |
|  |  |  |  |  |  | Implementation:  Fidelity, satisfaction | P-ESDM fidelity score, MBRS, tracking, questionnaire |
| Vismara et al. (2018) | ABA (ESDM) | Therapists (N/S) | VC-based parent-training/coaching: review of past practice, discussion, education, individual goal setting/planning  Self-directed module-based training via web platform: education (text/video), additional resources, caregiver-therapist communication | Less intensive intervention without P-ESDM content | 12 weeks   - 12 VC sessions: 90 min/session - Modules: self-paced | Child:  Social communication behavior | Video transcription |
|  |  |  |  |  |  | Implementation:  Fidelity, satisfaction | P-ESDM fidelity score, tracking, questionnaire |
| **CP or at risk of CP** | | | | | | | |
| Lima et al. (2023) | “Specific Task-Environment-Participation (STEP)”/  FCC | Physical therapist | VC-based parent-coaching: individual goal setting, consulting  Guided daily practice according to a provided booklet with logbook progress recording | Same intervention without individualized goal setting | 10 weeks   - 10 VC sessions: N/S - Self-practice (5 days/week): 30 min/day | Child:  Motor skills, participation | AIMS, YC-PEM |
|  |  |  |  |  |  | Caregiver/family:  Home environment | AHEMD-IS |
|  |  |  |  |  |  | Implementation:  Feasibility | Questionnaire |
| Pietruszewski et al. (2020) |  | Therapists (N/S) | VC-based parent-training/coaching with child using wearable device, movement sensor: content education, weekly follow-up assessment | Waitlist | 4 weeks   - 4 VC sessions: 15–45 min/day | Child:  Upper extremity motor capacity | Kinematic analysis, Baley scales |
| Schlichting et al. (2022) |  | Researchers (N/S) | VC-based parent-coaching with child: supervision, instant feedback on rehabilitation implementation  Guided daily practice with diary progress recording | N/A | 12 weeks   - 12 VC sessions: 60 min - Self-practice (4 times a week): 20 min/day | Child:  Motor skills | GMFM-88, AIMS |
|  |  |  |  |  |  |  |  |
| Svensson et al. (2024) | “Parent-Delivered Baby mCIMT Model”/mCIMT | Occupational therapist or physiotherapist | VC-based parent-coaching with child: supervised individualized rehabilitation practice  Web platform with education (text/video) and therapist communication  Guided daily practice of instructed activities, logbook progress recording on web platform | N/A | 6 weeks   - 6 VC sessions: N/S - Daily practice: 30 min/day | Child:  Hand motor | HAI |
|  |  |  |  |  |  | Implementation:  perception of the remote intervention | Questionnaire |
| **Other conditions** | | | | | | | |
| Akemoğlu et al. (2022) | “the i-PiCSS”/  NBDI | Researchers (N/S) | Self-directed, sequential module-based training: video tutorials, transcripts, pre-/postmodule quizzes  VC-based 1:1 coaching based on caregiver's achievement: individual feedback and Q&A | N/A | 10 weeks   - 5 modules: 15–25 min/module - 11–16 VC sessions: 15–20 min/session | Child:  Verbal & nonverbal responses, verbal & nonverbal initiations | Video coding |
|  |  |  |  |  |  | Implementation:  Fidelity, knowledge | Checklist, video coding, tracking, quizzes |
| Daczewitz et al. (2020) | “Parent-Implemented Communication Strategies (PiCS)”/  NDBI | Coach (N/S) | VC-based parent-training/coaching with the child: content explanation, individual planning, observation of parent-child interaction, instant feedback | N/A | 11 weeks   - 8 baseline sessions - 18 VC sessions: 15–25 min/session | Child:  Communication behaviors (responding, initiating) | Video coding |
|  |  |  |  |  |  | Implementation:  Fidelity, satisfaction, cost efficiency | Checklist, video coding, survey, interview, cost-benefit analysis |
| de Almeida Rodrigues et al. (2023) | “The Family-Professional Collaboration Model”/  FCC | Physical therapist | Parent-coaching via phone or video call: individual goal setting, daily adaptation planning, consulting  Guided daily practice without progress recording | N/A | 8 weeks   - 8 VC or phone sessions: 60 min/session | Child:  Motor performances, goal attainment | COPM, AIMS, PEDI-CAT, GAS |
|  |  |  |  |  |  | Caregiver/family:  Satisfaction with the child’s performances | COPM-satisfaction |
| Sgandurra et al. (2017) | “CareToy System” | Clinical/rehabilitative staff (N/S) | Self-directed system with individualized scenarios, modifications based on remotely monitored child’s condition | Standard intervention | 4 weeks   - Daily practice: 30–45 min/day | Child:  Motor skills, visual score | IMP, AIMS, Teller acuity cards |
| ABA: Applied Behavior Analysis; AHEMD-IS: The Affordances in the Home Environment for Motor Development-Infant Scale; AIMS: Alberta Infant Motor Scale; APSI: Autism Parent Screen for Infants; BCBA: Board Certification in Behavior Analysis; BST: Behavioral Skills Training; CBRS: The Child Behavior Rating Scale; CDI: MacArthur-Bates Communicative Development Inventory; COPM: Canadian Occupational Performance Measure; EMT: Enhanced Milieu Teaching; ESDM: Early Start Denver Model; FCC: Family-Centered Care; GARS: Guilliam Autism Rating Scale; GAS: Goal Attainment Scale; GMFM: Gross Motor Function Measure; HAI: Hand Assessment for Infants; IMP: Infant Motor Profile; M-CHAT-R/F: Modified Checklist for Autism in Toddlers, Revised, with Follow-Up; MBRS: Maternal Behavior Rating Scale; NBDI: Naturalistic Developmental Behavioral Interventions; PEDI-CAT: Pediatric Evaluation of Disability Inventory Computer Adaptive Test; P-ESDM: Parent-Early Start Denver Model; PML: Parent Mediated Learning; POEMS: Parent Observation of Early Markers Scale; PSI-SF: Parenting Stress Index 4; RBS-EC: Repetitive Behavior Scale for Early Childhood; RRBI: Restricted and Repetitive Behaviors and Interests; VC: Videoconferencing; YC-PEM: Young Children's Participation and Environment Measure | | | | | | | |
